# Supplementary material for: Assessment of three-dimensional RNA structure prediction in CASP15
Source: bioRxiv. 2023 Apr 26:2023.04.25.538330. Preprint. [Version 1] doi: 10.1101/2023.04.25.538330 (PMC10168427; doi:10.1101/2023.04.25.538330)
Supplement: Supplementary file 1 [file media-1.pdf]

Supplemental Information for "Assessment of three-dimensional RNA structure prediction in CASP15"

Rhiju Das[1,2,3,*,‡] https://orcid.org/0000-0001-7497-0972
Rachael C. Kretsch[2,*] https://orcid.org/0000-0002-6935-518X
Adam Simpkin[4,†] https://orcid.org/0000-0003-1883-9376
Thomas Mulvaney[5,6,†] https://orcid.org/0000-0002-4373-6160
Phillip Pham[1] https://orcid.org/0000-0002-0240-6384
Ramya Rangan[2] https://orcid.org/0000-0002-0960-0825
Fan Bu[7,8] https://orcid.org/0009-0000-1903-2270
Ronan Keegan[4,9] https://orcid.org/0000-0002-9495-0431
Maya Topf[5,6] https://orcid.org/0000-0002-8185-1215
Daniel Rigden[4] https://orcid.org/0000-0002-7565-8937
Zhichao Miao[10,11,‡] https://orcid.org/0000-0002-5777-9815
Eric Westhof[12,‡] https://orcid.org/0000-0002-6172-5422

[1]Department of Biochemistry and [2]Biophysics Program, Stanford University School of Medicine, CA USA, [3]Howard Hughes Medical Institute, Stanford University, CA USA, [4]Institute of Systems, Molecular & Integrative Biology, The University of Liverpool, UK, [5]Centre for Structural Systems Biology (CSSB), Leibniz-Institut für Virologie (LIV) and [6]University Medical Center Hamburg-Eppendorf (UKE), Hamburg, Germany, [7]Guangzhou Laboratory, Guangzhou International Bio Island, Guangzhou 510005, China, [8]Division of Life Sciences and Medicine,University of Science and Technology of China, Hefei 230036, Anhui, China, [9]Life Science, Diamond Light Source, Harwell Science, UK, [10]GMU-GIBH Joint School of Life Sciences and [11]The Guangdong-Hong Kong-Macau Joint Laboratory for Cell Fate Regulation and Diseases, Guangzhou Laboratory, Guangzhou Medical University, and [12]Architecture et Réactivité de l'ARN, Institut de Biologie Moléculaire et Cellulaire du CNRS, Université de Strasbourg, F-67084, Strasbourg, France. [*]Equally contributing authors. [†]Equally contributing authors. [‡]Correspondence to: rhiju@stanford.edu, miao_zhichao@gzlab.ac.cn, and eric.westhof@ibmc-cnrs.unistra.fr.

This Supplemental Information file contains one Supplemental Table and six Supplemental Figures.

Model	Best RMSD[a]				Model 1 RMSD[b]				Best DI[c]				Model 1 DI[c]				Best INF[d]				Model 1 INF[d]			
Rank	1st	2nd	3rd	sum[e]	1st	2nd	3rd	sum[e]	1st	2nd	3rd	sum[e]	1st	2nd	3rd	sum[e]	1st	2nd	3rd	sum[e]	1st	2nd	3rd	sum[e]
TS232	6	2	0	22	5	0	2	17	6	2	0	22	6	0	1	19	6	1	1	21	3	3	0	15
TS287	1	2	2	9	1	3	2	11	1	3	2	11	2	5	0	16	2	0	3	9	4	2	0	16
TS128	1	1	2	7	2	1	0	8	1	0	1	4	1	1	1	6	0	0	0	0	0	0	0	0
TS081	0	1	1	3	2	1	1	9	0	1	3	5	1	3	1	10	2	2	2	12	2	1	2	10
TS229	2	0	1	7	0	0	0	0	2	0	0	6	0	0	0	0	0	0	0	0	0	0	0	0
TS416	0	2	2	6	0	1	0	2	0	1	1	3	0	1	0	2	0	0	0	0	0	1	0	2
TS239	2	0	0	6	0	0	0	0	2	0	0	6	0	0	0	0	0	0	0	0	0	0	0	0
TS439	2	0	0	6	0	0	0	0	2	0	0	6	0	0	0	0	0	0	0	0	0	0	0	0
TS110	1	0	0	3	0	0	0	0	1	0	0	3	0	0	1	1	0	0	0	0	0	0	0	0
TS285	1	0	0	3	1	0	0	3	1	0	0	3	1	0	0	3	0	0	0	0	1	0	0	3
TS456	0	1	0	2	0	1	0	2	0	1	0	2	0	1	0	2	0	0	1	1	0	0	0	0
TS054	0	1	0	2	0	1	0	2	0	1	0	2	1	0	0	3	0	1	1	3	0	0	2	2
TS347	0	1	0	2	0	1	0	2	0	1	0	2	0	1	0	2	0	0	1	1	0	0	0	0
TS489	0	0	0	0	1	0	1	4	0	0	0	0	0	0	1	1	0	0	0	0	0	0	0	0
TS470	0	0	0	0	1	0	1	4	0	0	0	0	0	0	1	1	0	0	0	0	0	0	0	0
TS227	0	0	0	0	0	1	0	2	0	0	0	0	0	0	0	0	0	0	0	0	0	0	0	0
TS147	0	0	0	0	0	1	0	2	0	0	0	0	0	0	0	0	0	0	0	0	0	0	0	0
TS119	0	0	0	0	0	1	0	2	0	0	0	0	0	1	1	3	0	1	1	3	1	0	0	3
TS325	0	0	0	0	0	1	0	2	0	1	0	2	0	1	0	2	0	0	1	1	0	0	0	0
TS392	0	0	0	0	0	1	0	2	0	0	0	0	0	0	0	0	0	0	0	0	0	1	0	2
TS434	0	0	0	0	0	1	0	2	0	0	0	0	0	0	0	0	1	1	0	5	0	0	1	1
TS035	0	0	0	0	0	0	0	0	0	1	0	2	0	0	0	0	0	1	1	3	0	0	2	2
TSR01	0	0	0	0	0	0	0	0	0	0	2	2	0	0	1	1	0	0	1	1	0	1	0	2
TS125	0	0	0	0	0	0	0	0	0	0	1	1	0	0	1	1	0	1	0	2	0	0	1	1
TS235	0	0	0	0	0	0	0	0	0	0	0	0	0	0	1	1	0	0	0	0	0	0	1	1
TS076	0	0	0	0	0	0	0	0	0	0	0	0	0	0	1	1	0	0	0	0	0	0	0	0
TS444	0	0	0	0	0	0	0	0	0	0	0	0	0	0	1	1	0	2	0	4	0	1	1	3
TS248	0	0	0	0	0	0	0	0	0	0	0	0	0	0	0	0	0	0	1	1	0	0	1	1
TS185	0	0	0	0	0	0	0	0	0	0	0	0	0	0	0	0	0	0	0	0	1	0	0	3

[a] best RMSD reached for all the models submitted
[b] RMSD reached by the model ranked #1 by the group
[c] Same as [a,b] for Deformation Index (DI)
[d] Same as [c,d] for Interaction Network Fidelity (INF).
[e] For each group, the number of times the submitted models were ranked 1st, 2nd, or 3rd were counted and the weighted sum indicated in SUM (with a weight of 3 for 1st, 2 for 2nd and 1 for 3rd ranks).

Supplementary TABLE 1. Scores reached by the modeling groups using the different RNA-Puzzles metrics and assessment processes as indicated in the column Model.

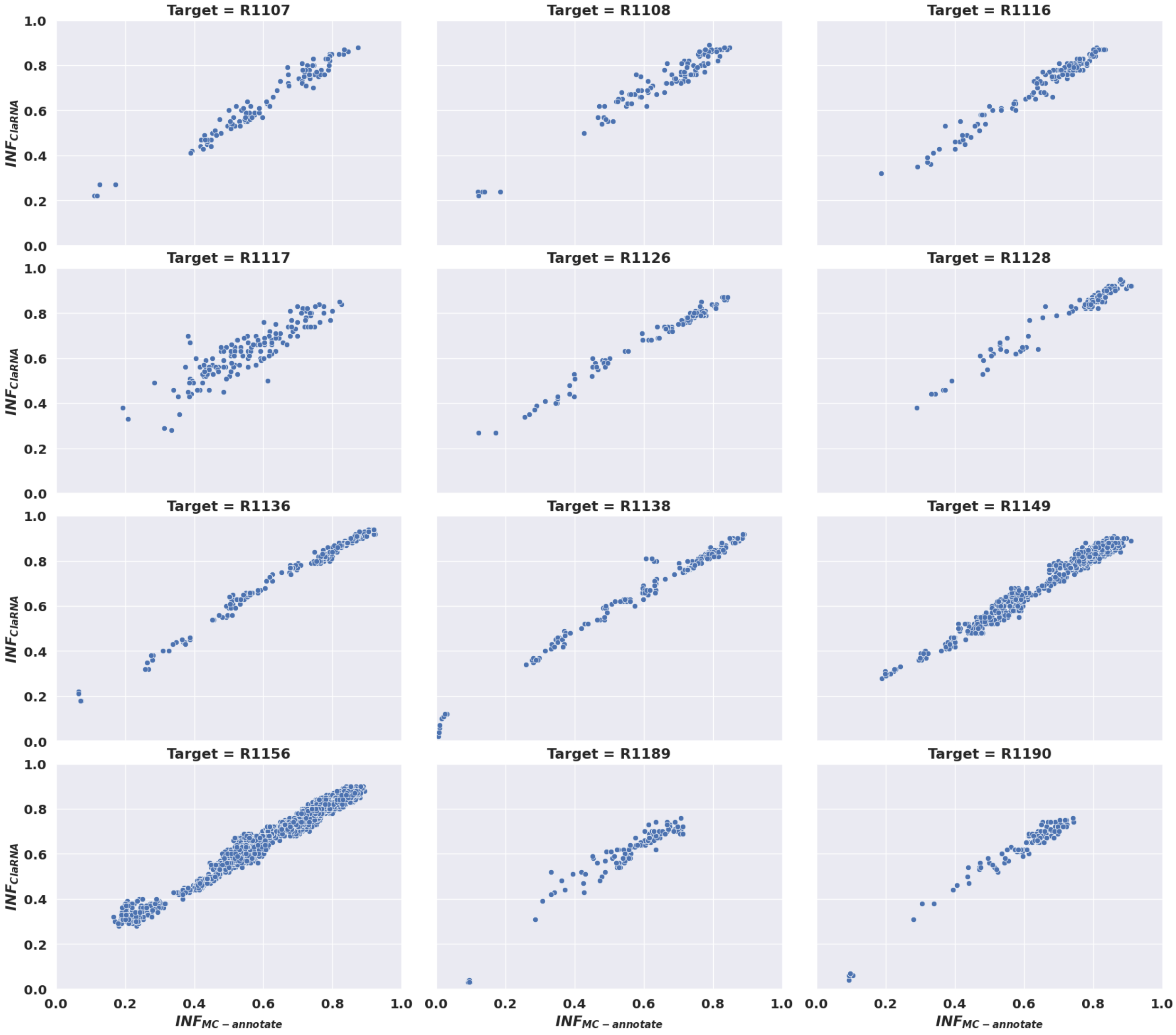

Supplemental Figure 1. Comparison of INF scores calculated using MC-annotate or ClaRNA.

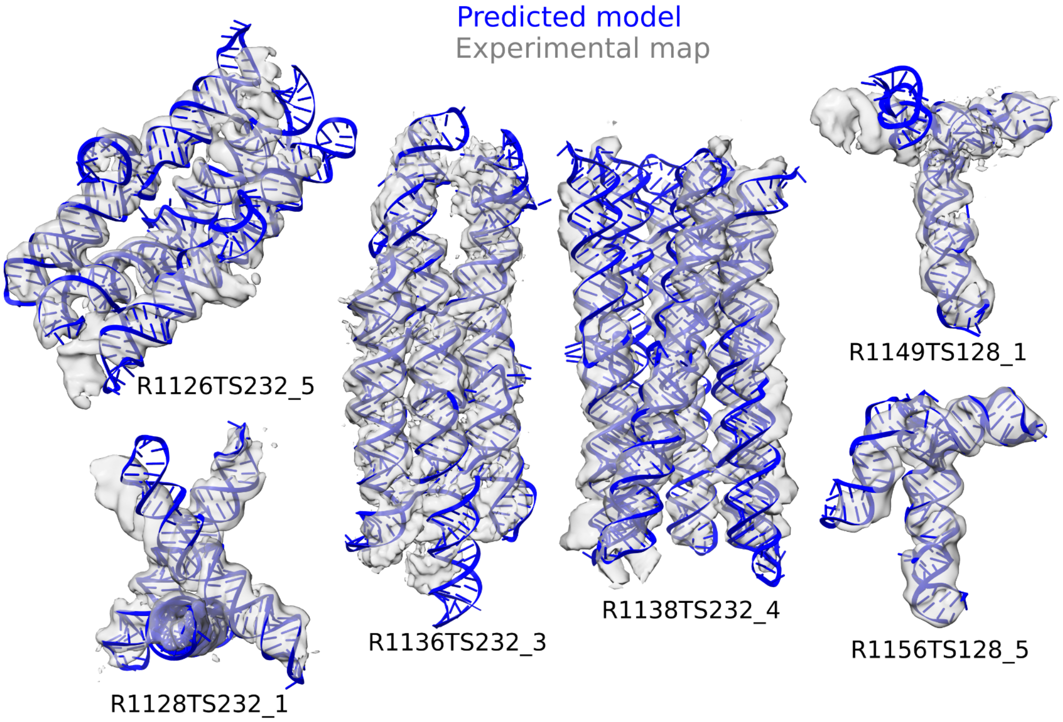

Supplemental Figure 2. The best fitting, by Z_{EM}, predicted model (blue) fit into an experimental cryo-EM map for each target (grey).

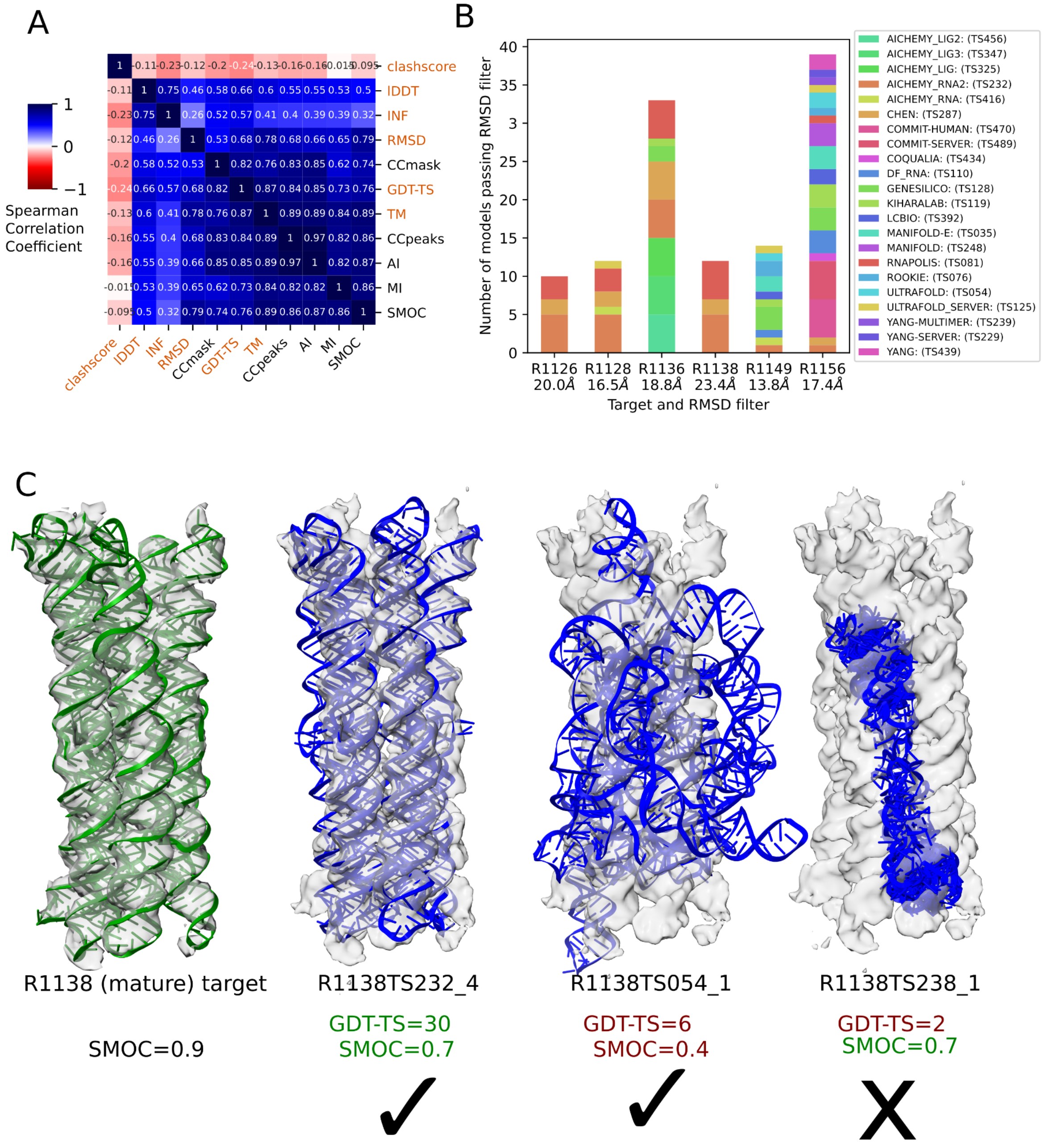

Supplemental Figure 3. (A) the Spearman correlation between metrics used in Z_{RNA}, Z_{EM}, as well as RMSD for all models passing the RMSD filter. (B) The number of models which have RMSD to target is less than the filter cutoff, so they were used in the final EM ranking. (C) An example of when EM metrics can be misleading with the target in green, the experimental map in grey and predicted models in blue. GDT-TS is reported as an example of model-to-model metric and SMOC as an example of map-to-model metrics. Green scores are seen as “good” while red are “poor” scores.

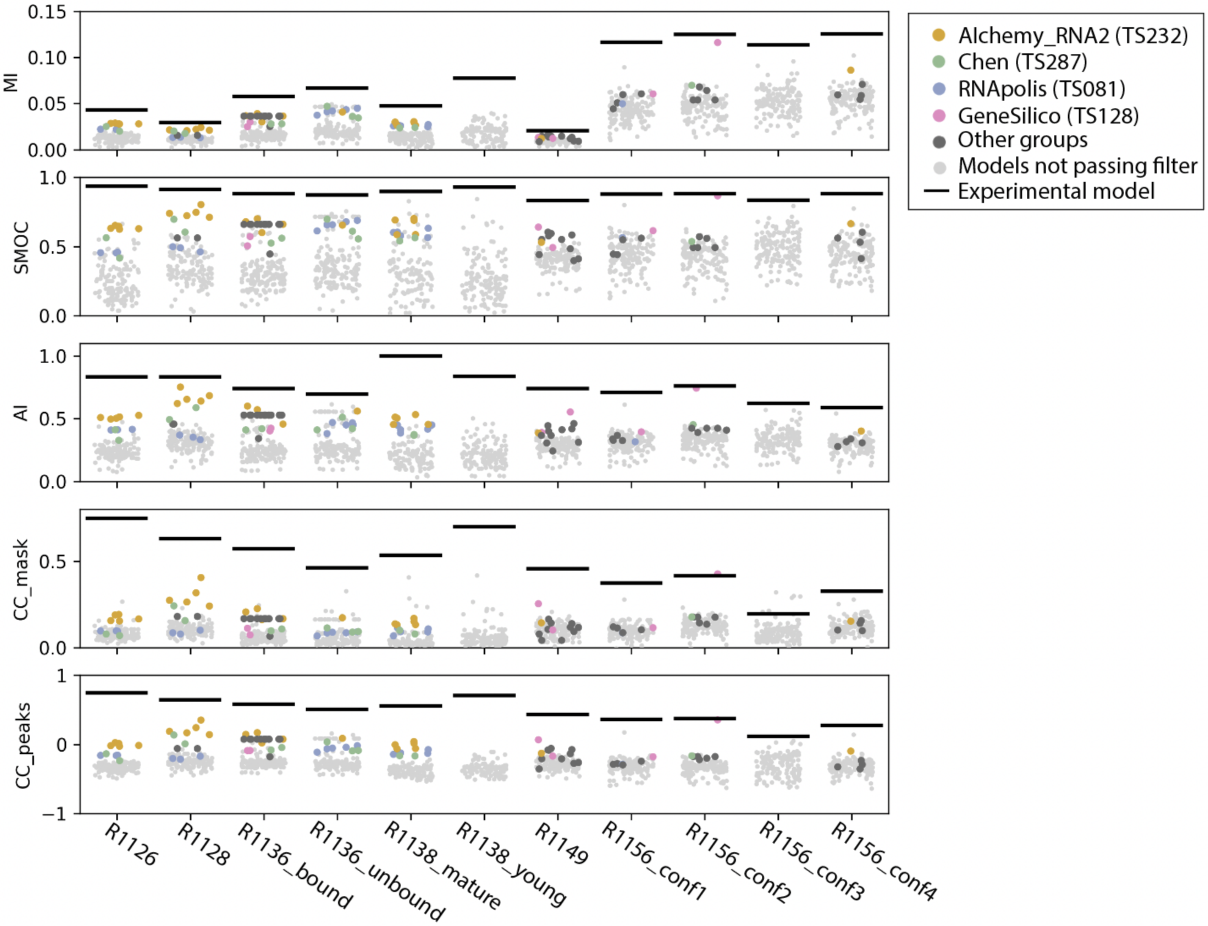

Supplemental Figure 4. EM metrics for all targets. Scores for all models submitted for all targets are depicted. Models passing the RMSD filter are indicated with larger dots, colored if submitted by top performing groups and dark gray otherwise. The black line indicates the EM metric scores for the experimentally determined model.

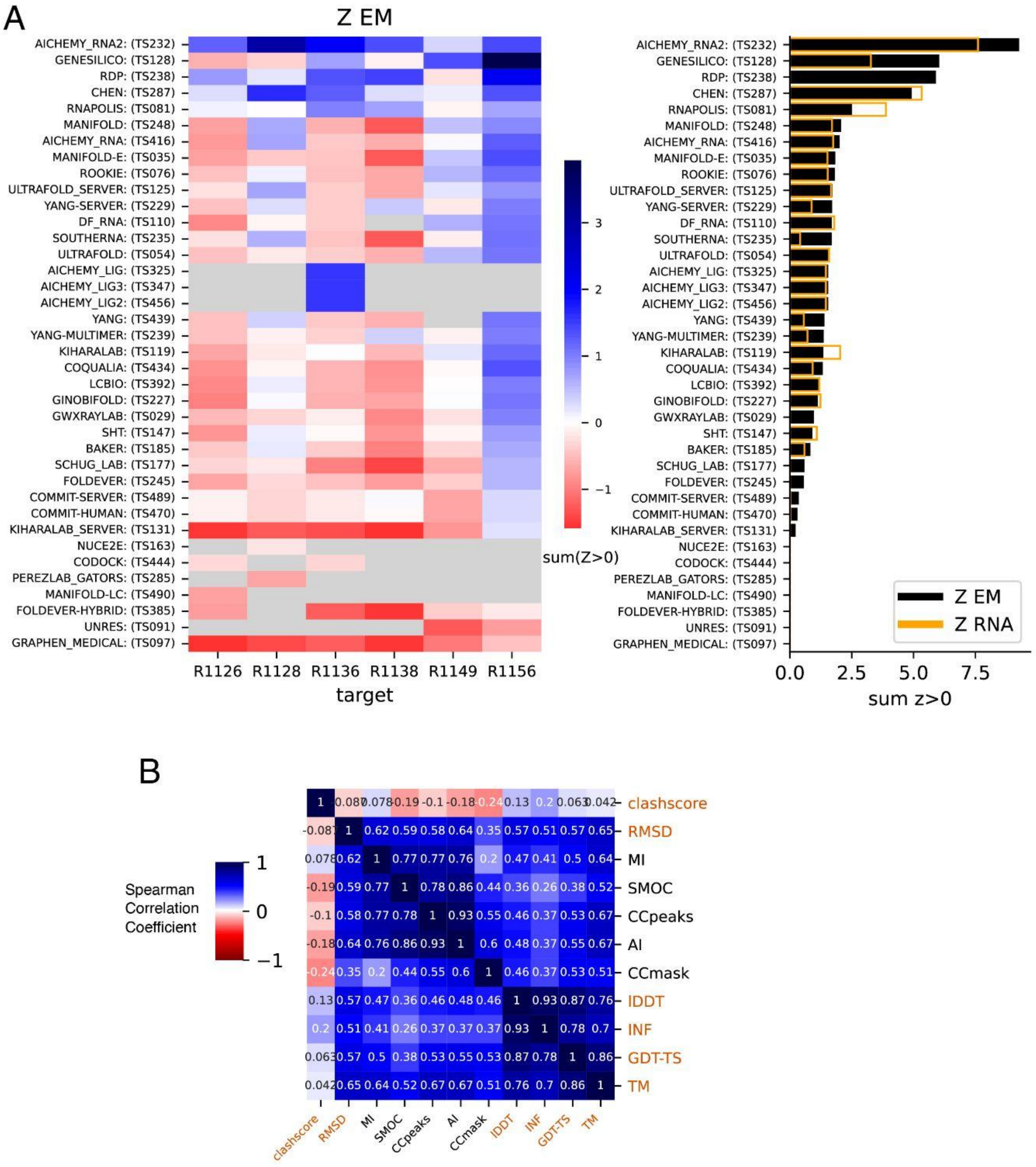

Supplemental Figure 5. (A) Z-scores for all models for RNA cryo-EM targets and ranking according to Z_{EM} (black), in orange are the Z_{RNA} scores for comparison. (B) the Spearman correlation between metrics used in Z_{RNA}, Z_{EM}, as well as RMSD for all models from cryo-EM targets.

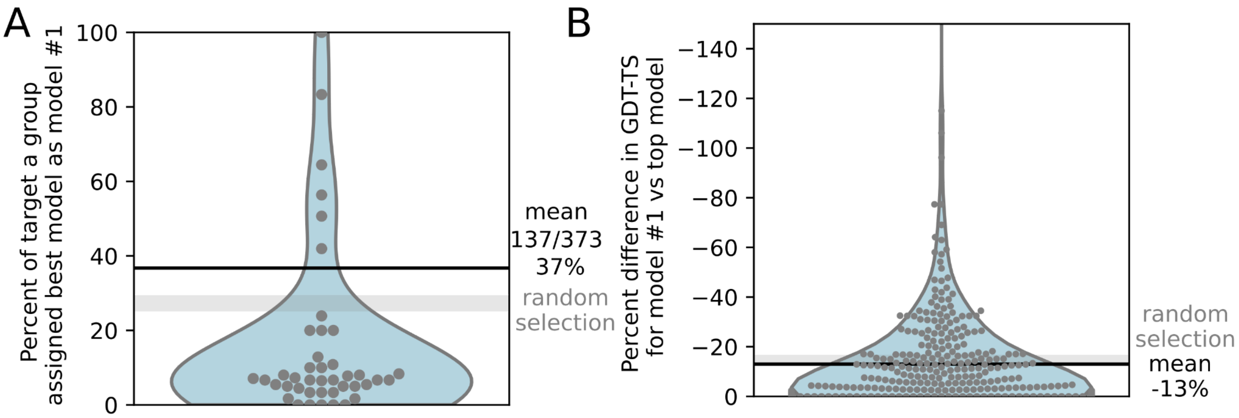

Supplemental Figure 6. Comparison of groups' top model by GDT-TS and the model they selected as model #1. (A) For each group, the percent of targets they participated in where their best model, by GDT-TS, was assigned as model #1. (B) For all targets and all groups, the percent difference in GDT-TS from model #1 to the top model for that group. The mean value for (A,B) is marked with a black line. If model #1 was selected randomly, the gray box marks the 95% confidence interval (1,000 and 10,000 bootstraps respectively).

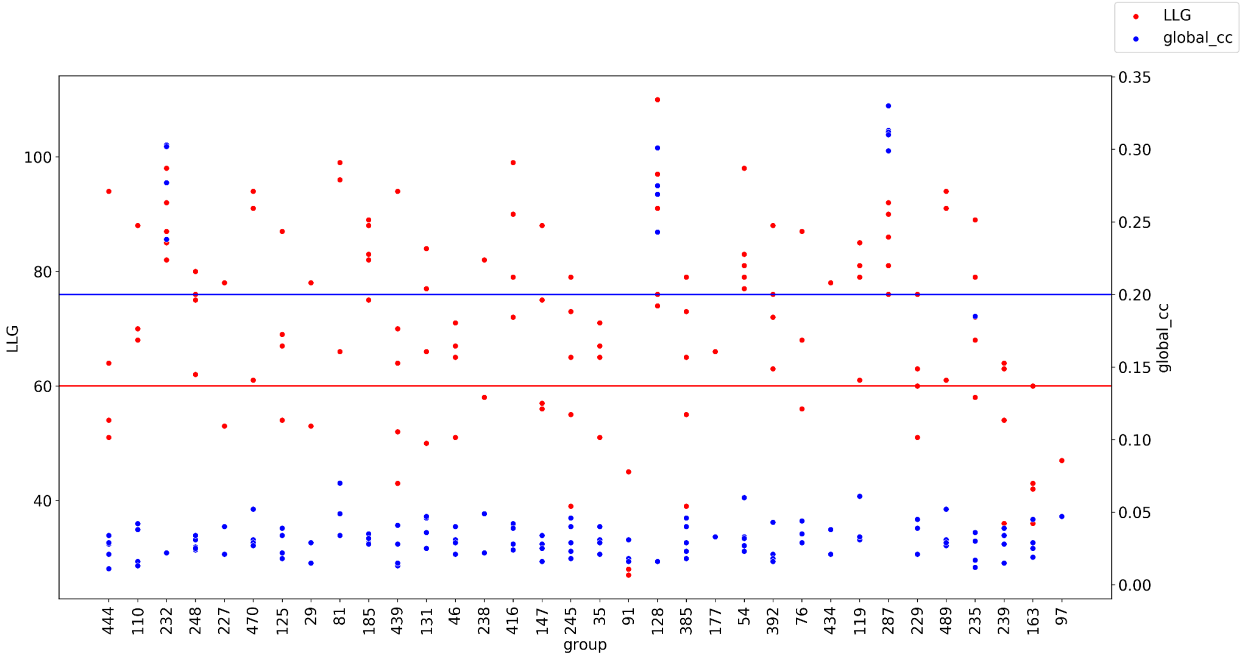

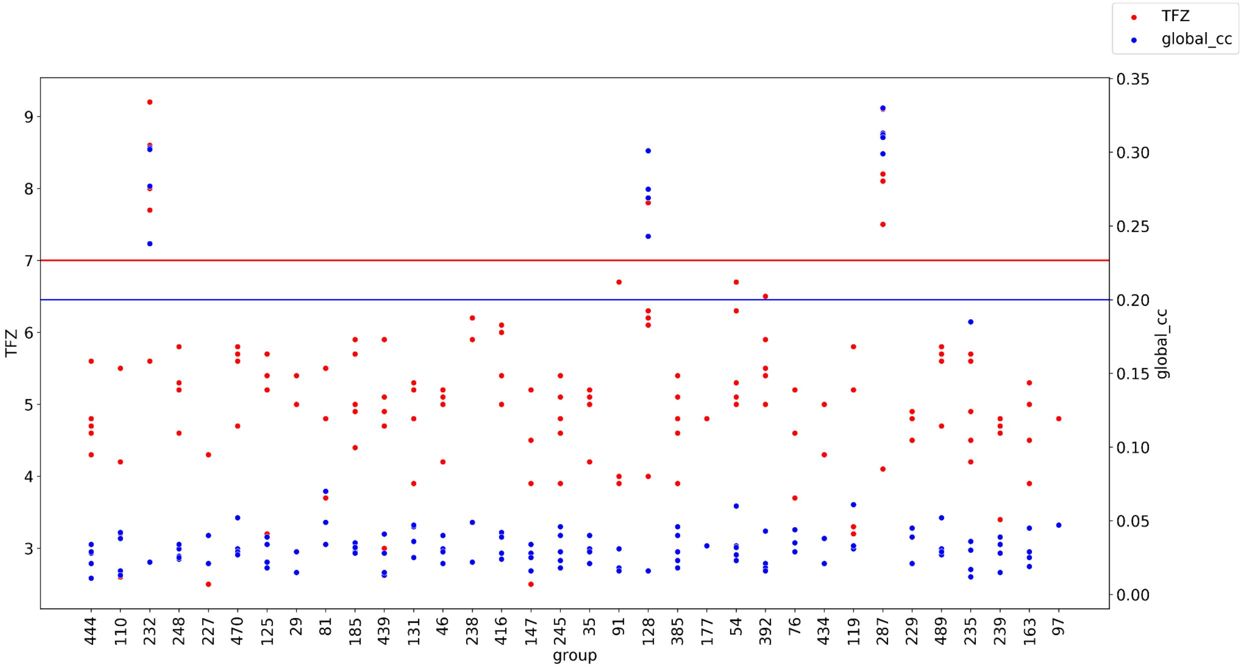

Supplemental Figure 7. Molecular replacement analysis of all groups for R1117. LLG (top) and TFZ (bottom) are plotted in red with a horizontal line at 60/7 respectively representing the normal criterion for successful placement. Global Map CC is plotted in blue with a horizontal line at 0.2 representing agreement between the placed model and the solved crystallographic structure.

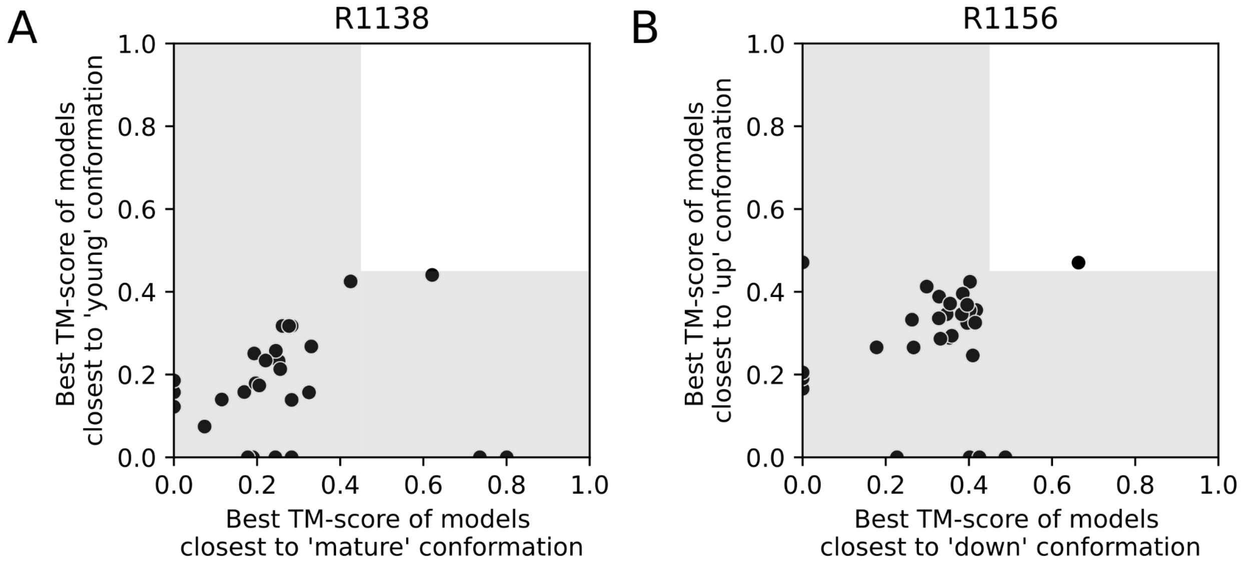

Supplemental Figure 8. Analysis how well groups modeled the multiple experimental models (A-B) All models are grouped by which experimental conformation the model is closest to, by TM-score, for the mature and young conformation R1138 (C) and 'up' and 'down' conformations for R1156 (D). For each group, the best TM-score for each group is plotted, with a score of 0 if no model was closest to that experimental conformation. Gray boxes indicate regions with TM-scores below expected for a correct topology (0.45), thus, the white area indicates groups that predicted two separate models topologically similar to the two experimental conformations.
